# 3D Culture of MSCs for Clinical Application

**DOI:** 10.3390/bioengineering11121199

**Published:** 2024-11-27

**Authors:** Qi Gao, Mehmet Sertac Cekuc, Yasemin Sude Ergul, Alexa K. Pius, Issei Shinohara, Masatoshi Murayama, Yosuke Susuki, Chao Ma, Mayu Morita, Simon Kwoon-Ho Chow, Stuart B. Goodman

**Affiliations:** Department of Orthopaedic Surgery, Stanford University, Stanford, CA 94304, USA; qigao7@stanford.edu (Q.G.);

**Keywords:** MSCs, 3D culture technology, in vivo trials

## Abstract

Mesenchymal stem cells (MSCs) play an important role in regenerative medicine and drug discovery due to their multipotential differentiation capabilities and immunomodulatory effects. Compared with traditional 2D cultures of MSCs, 3D cultures of MSCs have emerged as an effective approach to enhance cell viability, proliferation, and functionality, and provide a more relevant physiological environment. Here, we review the therapeutic potential of 3D-cultured MSCs, highlighting their roles in tissue regeneration and repair and drug screening. We further summarize successful cases that apply 3D MSCs in modeling disease states, enabling the identification of novel therapeutic strategies. Despite these promising applications, we discuss challenges that remain in the clinical translation of 3D MSC technologies, including stability, cell heterogeneity, and regulatory issues. We conclude by addressing these obstacles and emphasizing the need for further research to fully exploit the potential of 3D MSCs in clinical practice.

## 1. Introduction

Research on mesenchymal stem cells (MSCs) is an important area in regenerative medicine due to their high differentiation capacity, ability to promote tissue regeneration, and immunomodulatory properties [1,2]. MSCs are known for their differentiative potential in many cell lineages, including osteoblasts, chondrocytes, and adipocytes. In addition, MSCs help regulate the immune system and are used for cancer therapy and other immune diseases [3,4,5].

Traditional 2D cell cultures are limited in faithfully replicating complex tissue architecture, highlighting 3D culture approach for studying MSCs and their therapeutic potential. In 2D cultures, MSCs grow and expand on a flat substrate. Indeed, 2D-cultured MSCs have been extensively tested in clinical trials for various applications such as tissue regeneration, immune modulation, and nervous system disease [6,7]. A 2D culture provides a controlled environment to investigate cell responses after stimulation, which generates pre-conditioned MSCs. The pre-conditioned MSCs showed significant improvement of their immunoregulatory functions [8,9]. However, while useful, 2D cultures lack the necessary cell–matrix and cell–cell interactions to replicate the complex cellular interactions and microenvironment of native tissues. Recent advancements in 3D cell culture technologies facilitate a more accurate representation of physiological conditions. 3D-cultured MSCs have been extensively documented in the context of disease modeling and drug testing, showcasing their effectiveness in stimulating diseases such as osteoarthritis, osteoporosis, and other musculoskeletal disorders [10,11]. With trilineage differentiation abilities, 3D MSC models have been used to replicate osteogenesis and chondrogenesis processes in response to specific stimuli. MSCs also exhibit significant immunoregulatory functions, which allow them to not only modulate autoimmune diseases such as rheumatoid arthritis but also to regulate cancer progression [12]. Additionally, cytokines and exosomes from 3D MSCs were also reported to regulate wound healing [13,14]. Although 3D-cultured MSCs hold promise for regenerative medicine and tissue engineering, there are several challenges in applying 3D MSCs to clinical applications. MSCs are heterogeneous; their properties are determined in part by the location of origin, donor conditions, and culture environment [15,16]. Understanding these challenges benefits the integration of 3D MSC models into clinical practice.

In this review, we first introduce the methodologies employed in generating 3D MSCs, highlighting the aggregation method and various scaffolds, as well as their associated advantages and limitations. We then assess the therapeutic capacity of 3D MSCs, emphasizing their roles in tissue regeneration (trilineage differentiation abilities), disease modeling, and drug screening. Finally, we address the challenges and obstacles in 3D MSC technologies, including cell stability, standardization, cell heterogeneity, and regulatory considerations. We provide a comprehensive overview of the current state of 3D MSC research and its potential in clinical applications.

## 2. Methods Used for 3D Culture of MSCs

Although 3D cell culture models do not yet have a definitive and standard application protocol, structures such as spheroids and organoids/organ-on-chips are two prominent 3D structures that provide valuable platforms for a more in-depth study of biological processes.

### 2.1. Methods to Generate MSC Spheroids

MSC spheroids are three-dimensional spherical aggregates of MSCs. Several methods are commonly used to generate MSC spheroids, including low-attachment plates, magnetic levitation leverages, and the hanging drop method. Table 1 summarizes the key advantages and disadvantages of these methods employed for the generation of MSC spheroids.

Spheroids are formed when cultured in a low-adhesion environment, which prevents cells from attaching to the surface. For example, AggreWell™ technology provides a uniform approach where micro-wells are designed to facilitate cell aggregation [17]. The use of culture plates with ultra-low attachment surface is another approach [18]. Here, MSCs are seeded on the plates with ultra-low attachment properties and the cells are prevented from adhering to the plate. Thus, the cells move freely on the surface and form spheroids. Compared to other methods, this method is fast and practical and can be easily applied because it exhibits features similar to the standard 2D culture method. In the process of spheroid formation, efforts have focused on developing novel approaches to reduce cell adhesion. Successful reports include coating plates with agarose gel, chitosan, or poly(2-hydroxyethyl methacrylate) [18,19]. Employing a low-adhesion approach is straightforward and easy to manage, thus providing a convenient solution for researchers.

Magnetic levitation is another method in which magnetic particles are used to bring cells together and to form spheroids non-specifically with the help of magnets [20]. The high biocompatibility of this method allows cells to form spherical structures without the formation of undesirable inflammatory responses. For example, MSC spheroids were produced using magnetic nanoparticles to model bone marrow stem cell niches in vitro [21]. Interestingly, unlike other spheroid systems, cell viability in the inner region is consistent with that of the outer region. Applying magnetic levitation enhances cell–cell physical interactions. However, this approach demands the incorporation of extra magnetic equipment.

Generating MSC spheroids by the hanging drop method allows cell cultures with controlled sphere sizes. In this method, cells are released in a small volume liquid droplet and come together under the effect of gravity to form spheroids [22,23]. This technique encourages cells to grow and differentiate in a natural microenvironment by allowing cells to accumulate in a drop under the effect of gravity. This is a simple and reproducible method that supports cellular spheroid formation.

Besides these methods, recent scaffold-free approaches based on acoustic levitation have also shown that long-term culture of MSCs is possible [24,25]. Sound waves are used to manipulate the cell position without contact. Microfluidic chip, which has recently attracted attention, is another approach that allows MSCs to be passed through microchannels with a specific orientation in small-scale environments to form spheroids [26]. Rotary bioreactors are another innovative system that are designed to enhance MSC spheroid formation [27].

There are some general challenges in generating 3D spheroids. First is the possibility of low-efficiency or heterogeneous forms [28]. Some cell types may not aggregate effectively, requiring optimization. For example, 3D systems might not be superior to traditional 2D culture in the expansion of hematopoietic stem cells [29]. Larger spheroids may face nutrient and oxygen limitations, impacting cell viability. Some research takes advantage of the hypoxic environment in spheroids, such as cancer spheroids, to better understand tumor behavior and drug resistance. However, in other cases, it is crucial to avoid nutrient and oxygen limitations to ensure cell viability and overall function. When generating MSC spheroids, it is essential to optimize the culture medium and monitor the spheroid size and cell viability. By selecting the appropriate technique, researchers are able to utilize the specific benefits of MSC spheroids to further advancements in tissue engineering and regenerative medicine.

### 2.2. Generation of 3D MSC Culture Systems Using Scaffolds

The 3D culture of MSCs in scaffolds leads to the formation of organoid-like structures, known as organoids. These organoids consist of MSCs along with other organ-specific cells [30]. Organoids placed in a dynamic fluidic system are called organ-on-chips, which have recently attracted attention in examining dynamic cell interactions. Both organoids and organ-on-chip systems are currently superior and useful tools for creating in vivo-like environments that enable fundamental research on various tissues and organs compared to 2D cultures.

Organoids developed with MSCs allow cells to grow in and interact with ECM or scaffold fabricated using artificial or natural polymers. The common materials utilized for 3D MSC scaffolds are summarized in Table 2. For example, 3D-printed poly(ε-caprolactone) (PLA) is widely used to support MSCs growth and differentiation [31]. The PLA scaffold provides a biomechanically tunable framework for preclinical investigations of conditions such as critical-sized bone defects. However, the hydrophobicity of PLA significantly influences its interaction with MSCs. The incorporation of bio-ceramic nanocomposites is used to reduce the hydrophobicity of PLA, thus enhancing the induction and integration of the implanted scaffold [32]. Polymer polycaprolactone (PCL) is another commonly used scaffold that is used to investigate osteogenesis by MSCs. PCL nanofiber scaffolds enhance the migration, proliferation, and osteogenic differentiation of MSCs [33]. PCL scaffolds can be functionalized with natural polymers such as hyaluronan and ceramics such as tricalcium phosphate to explore osteogenic potential [34]. Chitosan scaffolds are widely investigated in cartilage repair as they promote chondrogenic differentiation of MSCs. In bone engineering, chitosan is frequently integrated with hydroxyapatite to increase mechanical properties. Applying transparent gelatin allows for straightforward observation of cellular behaviors. This characteristic enables researchers to monitor cell interactions and responses in real time. 3D-printed gelatin-based scaffolds were applied to explore the effect of pore size on MSC adipogenesis [35]. Scaffolds made from multiple materials provide an optimal environment for tissue regeneration. A chitosan–gelatin–chondroitin porous scaffold improves osteogenic differentiation of MSCs [36]. Furthermore, a modified collagen–glycosaminoglycan combined with biochemical supplementation enhanced MSC tenogenic, chondrogenic, and osteogenic differentiation [37].

Hydrogels represent a unique subclass of scaffolds characterized by their ability to swell in water. Commonly utilized materials include collagen, chitosan, hyaluronic acid, gelatin, and agarose. Although some hydrogels and scaffolds can be made from similar materials such as chitosan or collagen, they differ in their physical properties, applications, and manufacturing methods. Scaffolds are often designed to provide mechanical support, while hydrogels are typically soft. Furthermore, scaffolds are mainly fabricated using 3D printing, solvent casting, and electrospinning. Hydrogels are cross-linked by polymer chains to form gels. Hydrogels are also widely used in tissue engineering. Collagen gels have been widely used to promote the differentiation of MSCs, particularly for cartilage and tendon regeneration. For example, collagen scaffolds support the modeling of hematopoietic niches [38]. Collagen hydrogels incorporated with MSC spheroids facilitate neuronal differentiation while inhibiting inflammatory responses [39]. For bone engineering, collagen is often combined with ceramics or polymers to improve its mechanical properties. Chitosan-based hydrogel enhance the paracrine activity of MSCs that facilitates the treatment of spinal cord injuries [40]. Selecting the appropriate material and method is essential for further application.

Organ-on-chip integrated with MSCs mimics physical conditions in a dynamic process. The application of the joint-on-a-chip technology is a platform that simulates diarthrodial joint disorders in humans, and overcomes the limitations of many current models by using human cells for personalized medicine [41]. Bone marrow-on-chip is another example which incorporates the cellular components found in bone marrow including osteoblasts, endothelial cells, MSCs, and hematopoietic stem and progenitor cells [42]. A multi-organ chip with mature tissue niches linked by vascular flow enhances inter-organ interactions. MSCs also induce microvasculature formation in the microfluidic chips [43]. Bioreactor systems containing MSCs have shown great utility in understanding different disease mechanisms. MSCs can be encapsulated in gelatin methacrylate (GelMA) and cultured with separate flows of osteogenic, fibrogenic, and adipogenic environments to maintain the relevant tissue phenotypes and a common flow to provide tissue crosstalk [44]. This developed flow scheme can serve as a versatile in vitro platform for the effective examination of deteriorations in one or more tissues and the testing of drugs for personalized medicine.

Generating effective 3D culture systems presents several challenges. Appropriate 3D architecture that supports cell growth and differentiation is complex. Fortunately, 3D printing allows for customizing scaffold materials, porosity, and mechanical properties to meet the criteria of specific tissues or applications. A variety of methods are available for fabricating scaffolds with customizable parameters, including foam processing, solvent casting, electrospinning, and freeze-drying [45]. Spheroids/organoids generated by different techniques may result in varying characteristics. Standardizing protocols and accurately adjusting microenvironmental factors, including growth factors in the medium and pH stability, help generate consistent 3D MSCs. By addressing these factors, researchers can strengthen the reproducibility and reliability of spheroid-based assays, thereby enhancing application efficiency.

## 3. 3D Culture of MSCs Shows Therapeutic Capacities

**Figure 1:**
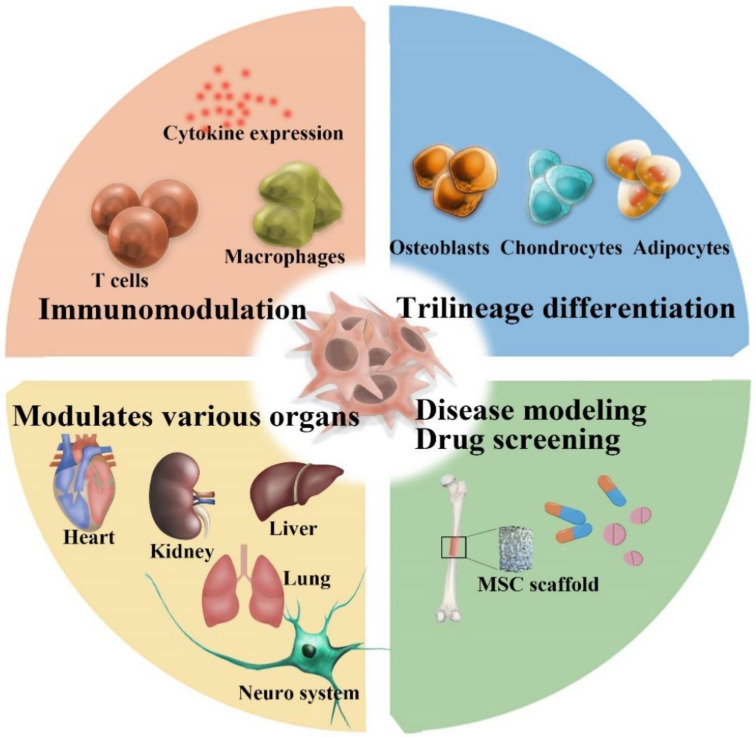
MSCs demonstrate therapeutic capacities, including trilineage differentiation, immunomodulation, organ modulation, and applications in disease modeling and drug testing.

As shown in Figure 1, MSCs have the capacity for differentiation into various lineages, especially osteogenic, chondrogenic, and adipogenic lines, as well as immunomodulatory and regenerative potential. Culturing MSCs in 3D structures makes it possible to study the differentiation, proliferation, and biological functions of stem cells more authentically.

### 3.1. Trilineage Differentiation (Osteogenesis, Chondrogenesis, Adipogenesis)

Bone regeneration is one of the most important targets of stem cell-based therapies in the fields of orthopedics and regenerative medicine. Identifying osteogenesis involves detecting alkaline phosphatase (ALP) activity and calcium deposition. In addition, qPCR is widely used to access the expression of osteogenic markers such as Runt-related transcription factor 2 (Runx2), osteocalcin, and osteopontin. MSC-encapsulated 3D culture environments are promising tools in the treatment of bone-related pathologies, as evidenced by the high expression of osteogenic markers Runx2, ALP, osteopontin, and collagen type I (COL-I) [46]. Specific signaling pathways and transcription factors regulate the osteogenesis process. Binding of Wnt ligands and Frizzled receptors leads to the accumulation of β-catenin in cytoplasm. Then, β-catenin translocates to the nucleus to activate osteogenesis. Spheroid MSCs had higher stemness than 2D monolayer MSCs, with rapid osteogenesis potential via the activation of the Wnt/β-catenin pathway [47]. The characteristics of the scaffold can direct the differentiation potential of MSCs that lead to increased bone formation. The biomechanical stiffness of the matrix modulates osteogenesis [48]. Implanting MSC-loaded scaffolds in bone defect models is a promising strategy to enhance bone healing [49]. The evaluation of in vivo bone formation can be employed via radiographic imaging, histological analysis, and biomechanical testing.

MSC chondrogenesis is a complex process and regulated by a variety of signaling pathways and transcription factors. Table 3 summarizes the key characteristics involved in MSC trilineage differentiation processes. A 3D culture system enhances MSC chondrogenesis. Platelet lysate in a 3D scaffold was shown to support MSC chondrogenesis [50]. Furthermore, a 3D bio-printed human cell-laden hydrogel construct also facilitated in vivo chondrogenesis [51]. Collagen II is a key component of ECM and is recognized as a hallmark of chondrogenesis. Using MSC-laden scaffolds, chondrogenesis in osteochondral defects was promoted by increasing collagen II production and suppressing IL-1β expression [52]. Mechanical stimulation promotes MSC chondrogenesis in 3D culture systems. The chondrogenic potential of 3D-cultured MSCs is indeed enhanced by mechanical stimulation such as compression, shear forces, and viscoelastic properties of the environment [53,54]. The mechanical loading regulated Rho-associated kinase (ROCK) pathway, TGF-β pathway.

Adipogenic differentiation is another promising area of research in regenerative medicine, particularly for applications in obesity, metabolic disorders, and tissue engineering. Spheroids demonstrated a greater enhancement of adipogenic differentiation in 3D cultures than in 2D cell cultures [55]. Utilizing the capacity of MSCs to produce the three main tissue types of joint components (bone, cartilage, and fat tissue) promotes the development of joint tissue organoids [56]. Differentiating MSCs in a 3D environment facilitates studying cell behavior and developing therapies.

### 3.2. Immunomodulation

3D culture of MSCs plays a crucial role in the regulation of immunomodulation. MSC spheroids transform MSCs into an immunomodulatory phenotype with anti-inflammatory properties [57,58]. Indeed, the immunomodulatory factors, including IDO, IL-10, LIF, ANG1, and VEGF, were found to be significantly increased in 3D-cultured Wharton’s jelly-MSCs [59]. 3D culturing infrapatellar fat pad-derived MSCs induces an enhanced immunomodulatory phenotype while effectively inducing resolution of inflammation/fibrosis of the synovium and fat pad [60]. These findings indicate 3D MSC spheroids have superior anti-inflammatory and immunomodulatory functions.

MSCs interact with immune cells such as macrophages, dendritic cells, and lymphocytes. For example, incorporation of MSCs into scaffolds amplified the immunomodulatory effects of macrophages. This paracrine interaction between MSCs and macrophages is regulated in part by 1,25-dihydroxyvitamin D3 [61]. In addition, MSC-laden scaffolds reverse the M1 macrophage phenotype, which is commonly associated with inflammatory responses and damage to tissues [62]. MSCs modulate the immune response involved in bone matrix development through the action of various cytokines that interact with both lymphocytes and macrophages [63]. MSCs mediated and reduced local and systemic inflammation through the triggering of an anti-inflammatory response. Thus, understanding the immune regulatory role of MSCs provides great advantages in tissue engineering applications.

### 3.3. 3D MSCs Modulate Various Organs

Studies on exploring neuroprotective effects of MSC spheroids/organoids have attracted attention. MSCs have the potential of differentiating into neuron-like cells, which offers promising opportunities for regenerative medicine. For example, MSCs have been differentiated into neuron-like cells using a composite scaffold with the induction of valproic acid induction [64]. Notably, MSCs harvested from adipose tissue are one of the candidates for use in neurological diseases as they exhibited significantly higher expression of neural markers and had a faster proliferation rate [65]. MSC spheroid-loaded collagen hydrogels simultaneously promote neuronal differentiation and suppress inflammatory reactions through the phosphatidylinositol 3-kinase (PI3K)/protein kinase B (AKT) signaling pathway [39]. Employing materials constitutes another strategy for enhancing neuroprotection by MSCs. A hybrid scaffold made by 3D reduced graphene oxide and collagen is utilized to promote the neural differentiation of MSCs [66]. MSCs promote nerve regeneration and regulate neuroinflammation. The use of spheroids formed from Schwann cells derived from umbilical cord blood MSCs significantly promoted the regeneration of peripheral nerves [67]. MSC spheroids alleviated neuropathic pain by modulating chronic inflammatory response genes. The expression levels of genes associated with inflammatory responses, specifically CCL11, IL-1A, and TNF, were significantly diminished following the administration of spheroids in contrast to monolayer treatments [68]. Spheroids generated from human placenta MSCs alleviate spinal cord injury in mice, leading to a significant increase in the release of anti-inflammatory and trophic factors including VEGF, PDGF, and FGF [69]. Therefore, transplanting 3D MSC/HUVEC spheroids may mitigate brain injury resulting from ischemic strokes [70].

3D MSCs also show therapeutic potential in other organs. MSCs increase alveolar differentiation in lung progenitor organoid cultures [71]. Injection of hybrid spheroids composed of MSCs and other cells into the renal cortex improves kidney function [72]. Bioengineered hepatic tissue has been constructed by culturing umbilical cord MSCs in porcine decellularized liver scaffolds [73]. 3D MSC cultures are also frequently preferred in heart tissue repair models and cardiovascular studies. The ability of MSCs to differentiate into cardiomyocytes has emerged as a significant subject of research in studies examining the regeneration of damaged myocardial tissue [74]. A 3D construct resembling liver tissue was fabricated by coculturing hepatocellular carcinoma cells (HepG2 cells), human umbilical vein endothelial cells (HUVECs), and MSCs [75]. 3D models currently implemented provide a critical platform for preclinical research that accelerates the integration of MSCs into clinical applications, especially in musculoskeletal, neurological, and cardiovascular tissue engineering, as well as in immunomodulatory therapies and chronic wound healing.

Although the application of 3D MSC-based cultures in the field of regenerative medicine has made tremendous progress in recent years, there are still significant challenges to overcome. Bone tissue is a complex structure consisting not only of osteoblasts, osteocytes, and osteoclasts, but also of immune and vascular cells and other elements located in the niche. Consequently, the regulation of metabolic processes presents significant challenges in the advancement of bone organoids. The enhancement of bone matrix development and mineralization can be achieved by co-culturing MSCs with immune cells, particularly in 3D environments [76]. These experimental models show considerable promise for disease research and regenerative medicine. The co-culture systems also contribute to illuminating the molecular mechanisms of interaction and communication that exist between distinct groups of cells. Maintaining the 3D culture system in the long term by providing a stable microenvironment of MSCs brings certain challenges. Effective strategies to monitor cell viability and functionality help ensure cells remain healthy and retain their important characteristics.

### 3.4. 3D MSCs for Disease Modeling and Drug Screening

The 3D culture of MSCs is of great interest, especially in modeling diseases and damage at the organ level, and in areas such as regenerative medicine and drug testing. The creation of 3D osteogenic models has shown higher sensitivity and biological significance than 2D monolayer cultures [77]. Investigating the differentiation of MSCs in the presence of a disease stimulus is crucial for mimicking disease processes. IL-1 serves as a commonly utilized cytokine that was incorporated into the 3D system to suppress chondrogenic development [78]. Mechanical stimulation plays a crucial role in the musculoskeletal system. An organ-on-chip model was also designed to study the role of mechanical stimuli on cartilage development and pathological onset. MSCs were cultured in fibrin gel in uBeat^®^ compression and tension platforms and mechanically stimulated to investigate the impact of mechanical stimuli on healthy cartilage development [79]. The combination of MSC differentiation and drug stimulation allows for the creation of in vitro models that closely resemble pathological states. For example, joint-on-a-chip is used in modeling processes that involve chronic degenerative changes in multiple tissues such as both bone and cartilage [80,81]. Following MSCs differentiation, IL-1β was added to induce degradative response. A human osteochondral unit-on-a-chip model was developed and exposed to active thyroid hormone to simulate osteoarthritis. This model contributes to the translation from osteoarthritis risk genes towards novel therapies [82].

Patient-derived MSCs retain the unique genetic and phenotypic characteristics of the donor. The efficacy of drug screenings was significantly increased in MSCs derived from both acute myeloid leukemia patients and healthy donors when placed in a 3D bone marrow niche [83]. Pediatric MSCs from donor BM aspirates were harvested and cocultured with leukemia cells to model bone marrow. This model was applied for ex vivo drug testing for Down syndrome treatment [84]. Spheroid cultures comprising human osteoarthritic chondrocytes and donor-matched chondrogenically differentiated MSCs were utilized to evaluate the efficacy of anti-TNF-α and anti-IL-1β drugs in short-term culture settings [85].

Applications of 3D MSCs extend beyond musculoskeletal diseases to areas such as cancer research and cardiovascular disease. An organoid-on-a-chip based on MSCs was designed to predict immunotherapy responses of hepatocellular carcinoma patients. Patients’ responses to anti-PD-L1 drugs were predicted using this technology [86]. A heart-on-a-chip platform capable of continuous non-invasive monitoring of the active force and passive tension was developed for disease modeling and drug development [87].

Several challenges affect the reliability of 3D MSC disease modeling. Accurately mimicking the disease microenvironment in vitro can be difficult, as many diseases involve complex interactions with various cell types and extracellular matrix components. Disease states involve dynamic changes making it challenging in consistently replicating these conditions in 3D cultures. Visualizing and analyzing cellular behavior in 3D cultures requires advanced imaging techniques, which are more complex than those used for 2D cultures, especially in the case of non-transparent materials. Evaluating cell viability, proliferation, and differentiation in 3D systems are also challenging due to the intricate architecture. Continued research into scaffold design and culture methods for accurately simulating disease microenvironments will enhance the effectiveness of these models for studying diseases.

## 4. Secretome of MSCs Cultured in 3D System

The role of growth factors, cytokines, and exosomes in the context of 3D culture systems for MSCs is crucial for regulating their behavior, enhancing their therapeutic potential, and facilitating tissue regeneration.

### 4.1. Growth Factors/Cytokines

MSCs secrete cytokines, chemokines, and growth factors to enhance cellular communication and improve immunomodulation. The synthesis of immunoregulatory cytokines such as IL-8, IL-6, IL-10, and IL-1β by MSCs in the context of closed proximal humerus fractures is essential for regulating inflammation and promoting tissue repair [88]. Increased secretion of anti-inflammatory markers was observed in MSC spheroid when compared with 2D culture [89]. MSCs were found to communicate with circulating leukocytes through a synergistic increase in FGF protein in a physiological flow system [90]. MSC-conditioned media also regulated macrophage polarization via cytokine and growth factor production [91]. More than 41 cytokines and growth factor production are involved in the regulation, such as IL-3, IL-8, and TGF-alpha. Furthermore, MSCs respond to the local microenvironment and change cytokine expression. In the context of cyclic strain, MSCs demonstrated a significant rise in the expression of anti-inflammatory cytokines (IL-10 and TGF-β1) [92]. 3D MSCs enhanced the level of proangiogenic factors, especially IL-6, vascular endothelial growth factor A (VEGFA), and IL-8, thereby promoting wound healing in a mouse model of burn injury [93].

Cytokines and growth factors also modulate the function of MSCs. IL-17 was found to modify the MSC niche to promote osteogenesis in collaboration with osteocytes [94]. The application of heparinized collagen biofabric enabled the controlled release of TGF-β, thereby influencing chondrogenesis by MSCs [95,96]. Interaction between MSCs and cytokines is a complex and dynamic relationship that significantly influences MSC functions. Exploring these interactions advances the therapeutic potential of MSC in regenerative medicine.

### 4.2. Exosomes

Extracellular vesicles play a significant role in cellular communication. Exosomes ranging from 30–150 nm enclosed in a lipid bilayer are the major population of extracellular vesicles released by MSCs [97]. Exosomes facilitate intercellular communication by transferring lipids, proteins, and nucleic acids [98] that support processes such as cell proliferation, survival, and differentiation.

Exosomes derived from 3D-cultured MSCs showed therapeutic properties. MSC exosomes can modulate immune responses. For example, exosomes from 3D-cultured MSCs alleviate knee osteoarthritis by promoting M2 macrophage polarization through miR-365a-5p and suppressing TLR2/Myd88/NF-κB signaling pathway [99]. In addition, exosomes from 3D MSCs demonstrated improved therapeutic effects in the treatment of periodontitis and experimental colitis, as well as the restoration of the Th17 cell to Treg cell balance within the inflamed periodontal area. [100]. Exosomes enhance tissue regeneration directly. Exosomes secreted from adipose-derived MSC promote tenogenic differentiation of tendon stem cells by activating SMAD1/5/9 and SMAD2/3 [101]. A gelatin methacryloyl microneedle loaded with 3D-MSC-exosomes provided ischemia-reperfusion in vascular models [102]. Thus, exosomes serve as important mediators of intercellular signaling.

The therapeutic potential of MSC is modulated via exosome secretion. During bone remodeling, exosomes overcome cellular barriers to communicate with neighboring cells and exchange information. Specifically, exosomes in bone promote angiogenesis and osteogenesis in injured areas. Exosomes derived from macrophages, particularly M1 macrophages, facilitate osteogenesis through cellular signaling and stimulation of osteoblast differentiation in bone marrow MSCs [103]. Exosomes also affect bone remodeling by decreasing osteoclast formation and stimulating osteoblast proliferation, leading to an imbalance [104,105]. Exosomes secreted by epithelial cells facilitate angiogenesis, modulate immune responses, encourage osteogenesis, and regulate downregulate osteoclast activity. While their therapeutic applications require further research, ongoing clinical trials are demonstrating promising results.

Exosomes offer minimally invasive therapies with reduced potential for adverse effects to the patient [106]. Despite the completion of clinical trials, there are currently no approved exosome-based therapies. Challenges regarding exosome collection and isolation methods persist, for example in the isolation of impure exosomes. Confirmation of exosome purity and functions requires multiple assays. Utilizing both ultracentrifugation and size-selective chromatography may help improve purity. Identifying effective factors in exosomes and exploring the downstream mechanisms are crucial for clinical therapies. Storage and dosage determination are other important aspects. pH value and temperature significantly influence the stability, functionality, and effectiveness of cytokines and exosomes. Future research should focus on optimizing scaffold materials and culture conditions to modulate the secretory capabilities of MSCs.

## 5. Challenges in Clinical Applications of 3D MSCs

Even though MSCs are known for their increased cellular and regenerative potential in clinical trials, there are many challenges, including the low quantities often harvested and difficulty in isolation. Other factors such as donor age and even culture conditions may impact the quality of the MSCs.

### 5.1. MSC Stability and Heterogeneity

Donor age and general health also influence the functional properties of MSCs. Age-related changes in MSCs are noteworthy, especially for regenerative medicine. MSCs derived from older patients exhibit a reduced ability to differentiate into multiple cell types. The reduced differentiation capacity observed in aged MSCs can be attributed to diminished cellular signaling pathways associated with p53 expression [107,108]. The migration capacity of these MSCs to injury sites is notably diminished, thereby affecting their effectiveness in the process of tissue repair. This reduction is linked to a decrease in cell migration signaling pathways and a lower expression of cytokines [107]. The therapeutic efficiency of MSCs is also altered by disease-related changes, especially in immune and inflammatory diseases. Table 4 summarizes the MSC functions derived from various donors under different conditions. In osteoarthritis-affected joint tissue, inflammatory cytokines contribute to the impairment of MSC functionality, which in turn disrupts the repair mechanisms of cartilage repair. MSCs from patients with advanced osteoarthritis show reduced chondrogenic and adipogenic activity [109]. In addition, obesity induces dysfunction and early senescence in adipose tissue-derived MSCs, as evidenced by the expression of the genes p16, p53, IL-6, and MCP-1 [110]. After allogeneic hematopoietic stem cell transplantation, there is an increase in ROS and p53 levels within MSCs, which contributes to the impairment of graft function [111].

**Figure 2:**
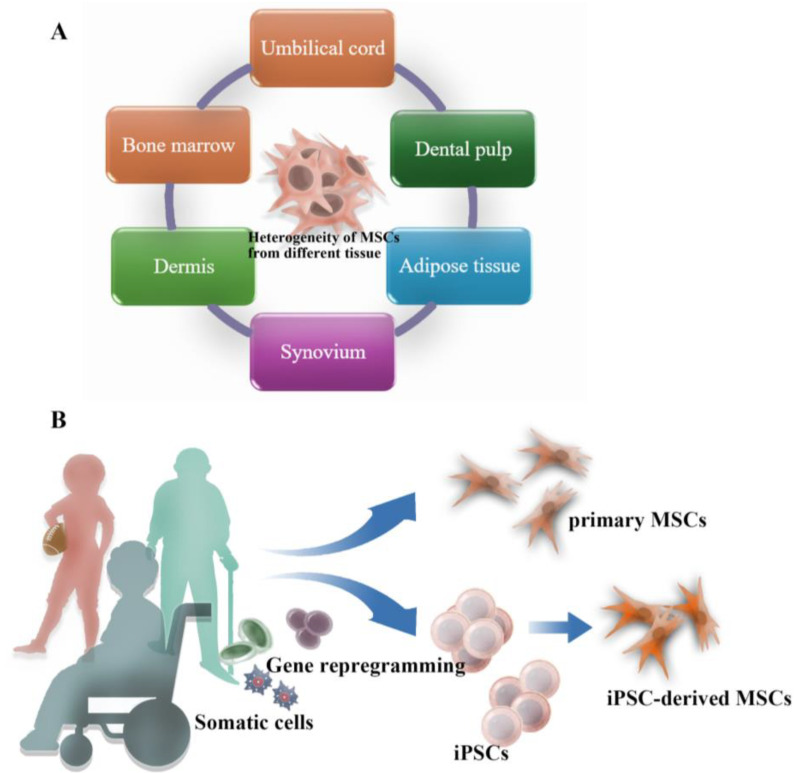
Heterogeneity of MSCs across different sources. (**A**) MSCs can be isolated from various tissues, including bone marrow, adipose tissue, umbilical cord, and others. (**B**) iPSCs provide an alternative to traditional stem cell sources.

MSCs from different anatomical locations exhibit significant heterogeneity in terms of their functional characteristics, and biological responses, as shown in Figure 2A. MSCs can be collected from distinct types of tissue including bone marrow, adipose tissue, umbilical cord, and dental pulp. MSCs differ in function depending on their location of harvest. For example, MSCs obtained from bone marrow are considered as the gold standard for osteogenic differentiation. In comparison, MSCs sourced from adipose tissue do not demonstrate the same level of effectiveness in osteogenic differentiation, despite exhibiting a higher rate of proliferation [112,113,114,115]. Surface markers were utilized to isolate specific subpopulations of MSCs to investigate their unique attributes [114]. However, the osteogenic potential observed in 2D cell cultures is not always consistent with the outcome from 3D systems. CD271-positive enrichment of a population is not beneficial for osteogenesis when the cells are seeded in 3D scaffolds [115]. Single-cell RNA sequencing analysis suggests that spheroid culture of heterogeneous MSCs synchronized the cells into a uniform cell population with increased levels of immunosuppressive genes and growth factors [58]. The heterogeneity of MSC cells may explain the variation in clinical performance and inconsistent results. The variability of donors, including age and health status, poses challenges in therapeutic efficiency and potency, thus leading to difficulties in clinical application.

**Table 4:** Summary of MSCs derived from different donors under varying conditions.

Donor Condition	MSC Source	MSC Functions
Osteoarthritis	Bone marrow aspirates obtained from the iliac crest or from the tibia/femur during joint surgery	Reduced proliferative capacity. Reduced chondrogenic and adipogenic activity [109].
Osteonecrosis and Osteoarthritis	Bone marrow of the proximal femurs	Osteogenic differentiation ability of MSCs in patients with alcohol-induced and idiopathic ON was significantly reduced compared with that in patients with OA [116].
Rheumatoid Arthritis and Osteoarthritis	Bone marrow aspirate from the femoral shaft	Demonstrated similar chondrogenic potential as MSCs isolated from healthy donors [117].
Corticosteroid-Induced Osteonecrosis	Bone marrow aspirates obtained from the iliac crest	Decreased cellular activity and osteogenesis ability [118].
Rheumatic Diseases	Subcutaneous abdominal fat	Inadequate immunoregulatory function [119].
Systemic Lupus Erythematosus	Bone marrow aspirate	Demonstrated early signs of senescence [120].
Graft-Versus-Host Disease	Bone marrow aspirate	Displayed normal immunophenotype and plasticity. Displayed normal immunosuppressive capabilities [121].
Obesity	Subcutaneous abdominal fat	Demonstrated early senescence program [110].
B-Cell Acute Lymphoblastic Leukemia	Bone marrow aspirate	Display normal immunosuppress T-cell responses but do not compromise CD19-CAR T-cell activity [122].
Multiple Sclerosis	Bone marrow aspirate	Reduced proliferative capacity. Ex vivo premature ageing [123].
Adolescent Idiopathic Scoliosis	Bone marrow aspirate	Decreased osteogenic differentiation ability and alkaline phosphatase activity [124].
Myelodysplastic and Acute Myeloid Leukemia	Bone marrow aspirate	Displayed similar surface marker expression and efficient capacity to differentiate versus osteogenic and adipogenic lineage in vitro. Reduced proliferative potential [125].
“No-Option” Critical Limb Ischemia	Bone marrow aspirate	Reduced proliferative capacity [126].

### 5.2. iPSC-Derived MSCs

Induced pluripotent stem cells (iPSCs) can be generated by transfecting Yamanaka factors (Oct4, Sox2, Klf4, and c-Myc) to somatic cells, reverting them into pluripotent cells. Consequently, iPSCs can be differentiated into MSCs, as shown in Figure 2B. These iPSC-MSCs share many characteristics with native MSCs, including surface markers (such as CD73, CD90, CD105) and multilineage differentiation potential (e.g., into osteoblasts, chondrocytes, and adipocytes).

One main advantage of using iPSCs is their ability to create a generous supply of MSCs and their potential for self-renewal [127]. iPSC-derived MSCs from aged individuals develop a rejuvenation signature, thereby improving their efficacy in cell replacement therapy [128]. Also, iPSC cells have reduced immunogenicity, which decreases the risk of immune rejection and adds to their use in many fields such as in transplantation [129]. Indeed, compared with the donor-matched parental MSCs, iPSC-derived MSCs showed an enhanced secretion of paracrine cytokine/growth factors, as well as more potent immune suppression [130]. iPSC cells can be modified genetically to correct mutations in the diseased patient [131]. iPSCs were used to correct a deficiency in a 59-year-old female patient with frontotemporal dementia and parkinsonism who was carrying an R406W mutation in the microtubule-associated protein tau (MAPT) gene.

There are several challenges in applying iPSC-derived MSCs. First is safety concern. It is essential to ensure that iPSC-MSCs do not give rise to tumors or exhibit abnormal behaviors after transplantation. Second, developing consistent protocols for generating iPSCs and differentiating into MSCs is vital for reproducibility in research and clinical applications. For example, iPSC-derived MSCs exhibit sufficient osteogenic and chondrogenic capabilities, while demonstrating reduced adipogenic potential [132]. Comprehensive pre-clinical testing is necessary before application.

## 6. Future Perspectives and Conclusions

Several promising directions involving MSCs significantly enhance their clinical application in regenerative medicine. The first aspect is the enhanced bioprinting technique. For example, layer-by-layer 3D printing facilitates precise control of the spatial arrangement of different cell types and growth factors, which better replicates native tissue architecture. Second is the use of patient-derived MSCs for personalized medicine. iPSC-derived MSCs may enhance personalized medicine by increasing treatment efficacy and minimizing adverse effects. In addition, fabricating advanced biomaterials that not only demonstrate enhanced biocompatibility, biodegradability, and promote biological activities, but also provide mechanical support will further improve MSC functionality. Smart biomaterials such as time-controlled drug delivery systems in a time-dependent series will enhance tissue integration and regeneration. Future research should also focus on developing 3D co-culture systems that include other key cell types, such as endothelial cells and immune cells. These multicellular models can better simulate the complex interactions occurring in vivo, improving the predictive capacity of disease models and therapeutic outcomes.

The exploration of 3D culture of MSCs represents a transformative advancement in regenerative medicine. 3D culture systems enhance the viability, functionality, and therapeutic potential of MSCs compared to traditional 2D approaches. This review highlighted the promising applications of 3D-cultured MSCs in tissue regeneration, disease modeling, and drug screening, demonstrating their capacity to contribute to innovative therapeutic strategies. However, several challenges must be addressed to facilitate the clinical translation of these technologies. Concerns associated with scalability, standardization, and regulatory compliance remain critical barriers. Future research focused on improving bioprinting techniques, integrating advanced biomaterials, and developing personalized approaches will be pivotal in overcoming these obstacles.

## Figures and Tables

**Table 1:** Summary of the methods to generate MSC spheroids.

Method	Advantages	Limitations
Low-Adhesion Plates	Simple. Compatible with most laboratory equipment. Reproducible. Provides a single spheroid per culture system/well.	Suspension cells may not aggregate efficiently. Cells which require more extracellular matrix support may not aggregate efficiently. Not suitable for long-term culture.
Magnetic	Promotes spherical growth and cell aggregation without the need for physical scaffolds. Allows for controlled spheroid size. Promotes improved nutrient distribution.	Needs specialized equipment. Possible cytotoxic effects from magnetic beads.
Hanging Drop Method	Simple and low-cost. Does not require special equipment. Ease of use and high reproducibility. Minimizes the risk of contamination of collagen scaffolds with exogenous proteins.	Evaporation of the medium could lead to dehydration. The alteration of surface tension, which plays a vital role in sustaining the drop’s form, disrupts the spheroid culture.
Acoustics Levitation	Promotes spherical growth and cell aggregation without physical contact.	Not all cell types respond well to acoustic levitation. Requires specialized equipment.
Rotary Bioreactor or Rotational Methods	Provides equal nutrient and oxygen diffusion. It is possible to obtain larger spheroids. Allows to add other cell types, which increases the opportunities for creating more complex tissues.	Requires specialized equipment. There is a risk of cell clustering.

**Table 2:** Materials utilized for MSC organoids.

Method	Appropriate Systems/Organ System to Mimic	Advantages	Limitations
PCL (Polycaprolactone)	Widely used in load-bearing systems such as bone defects and cartilage.	Biocompatibility. Easy processability, printability and flexibility. Slow degradation properties.	Limits cell adhesion from hydrophobic structure. Long degradation may negatively affect tissue regeneration.
PLA (Polylactic Acid)	Widely used in skin, cartilage, and cardiovascular applications.	Faster biodegradation than PCL. Resorbable. FDA approved. Commendable printing capacity.	Weaker mechanical strength compared to PCL, which makes it restrictive for hard tissue applications. Low cell affinity. Acidic byproducts generated during degradation can cause inflammation. Hydrophobicity.
Collagen	Simulating soft tissues such as skin, tendons, and cardiovascular system. Used for bone regeneration when combined with hard biomaterials.	Promotes cell adhesion, differentiation, and migration. Easily cross-linked to modify mechanical properties. Major element of the natural bone extracellular matrix. High biocompatibility and biological activity.	Exhibits poor mechanical properties for load-bearing tissues. Degrades rapidly.
Gelatine	Widely used for wound healing and skin tissue models. Modified form (methacrylated) for bone and cartilage tissue.	High cell adhesion properties. Biodegradable and biocompatible. Easily modified through chemical or physical methods.	Lacks mechanical strength. Degrades rapidly in vivo.
Chitosan	Widely used to mimic tissues such as cartilage, bone and skin.	Promotes chondrogenesis. Reduces infection risk with antibacterial properties. Biocompatibility and biodegradability.	Poor mechanical stability. Hydrophobic structure limits water solubility and cytoskeletal interactions.

**Table 3:** Characteristics of MSC trilineage differentiation.

	Osteogenesis	Chondrogenesis	Adipogenesis
Transcriptional factors	Runx2 Nuclear factor-kappa B (NF-κB) Osterix (Osx)	SRY-box transcription factor 9 (Sox9) Runx2	Peroxisome proliferator-activated receptor gamma (PPARγ) CCAAT enhancer binding protein alpha gene (CEBPA)
Mark genes	Runx2 Oosteocalcin Osteopontin ALP	Sox9 Aggrecan COL2A1	Lipoprotein lipase (LPL) Adiponectin Perilipin 1 (PLIN1) CEBPA
Key signaling pathways	Bone morphogenetic protein (BMP) signaling Wnt pathway Transforming growth factor-beta (TGF-β) signaling Fibroblast growth factor (FGF) signaling Notch signaling	Transforming growth factor-beta (TGF-β) pathway Bone morphogenetic proteins (BMPs) Wnt pathway	Insulin signaling pathway Wnt signaling pathway Peroxisome proliferator-activated receptors (PPARs)
Histological staining	Alizarin red S staining ALP staining	Alcian blue staining Safranin O staining	Oil red O staining Sudan red staining
